# Med25 Limits Master Regulators That Govern Adipogenesis

**DOI:** 10.3390/ijms24076155

**Published:** 2023-03-24

**Authors:** Jasmine Saunders, Kunal Sikder, Elizabeth Phillips, Anurag Ishwar, David Mothy, Kenneth B. Margulies, Jason C. Choi

**Affiliations:** 1 Center for Translational Medicine, Department of Medicine, Thomas Jefferson University, Philadelphia, PA 19107, USA; 2 Cardiovascular Institute, Perelman School of Medicine, University of Pennsylvania, Philadelphia, PA 19104, USA

**Keywords:** LMNA, mediator complex, lipid accumulation, adipogenesis

## Abstract

Mediator 25 (Med25) is a member of the mediator complex that relays signals from transcription factors to the RNA polymerase II machinery. Multiple transcription factors, particularly those involved in lipid metabolism, utilize the mediator complex, but how Med25 is involved in this context is unclear. We previously identified Med25 in a translatome screen of adult cardiomyocytes (CMs) in a novel cell type-specific model of *LMNA* cardiomyopathy. In this study, we show that Med25 upregulation is coincident with myocardial lipid accumulation. To ascertain the role of Med25 in lipid accumulation, we utilized iPSC-derived and neonatal CMs to recapitulate the in vivo phenotype by depleting lamins A and C (lamin A/C) in vitro. Although lamin A/C depletion elicits lipid accumulation, this effect appears to be mediated by divergent mechanisms dependent on the CM developmental state. To directly investigate Med25 in lipid accumulation, we induced adipogenesis in *Med25*-silenced 3T3-L1 preadipocytes and detected enhanced lipid accumulation. Assessment of pertinent mediators driving adipogenesis revealed that C/EBPα and PPARγ are super-induced by *Med25* silencing. Our results indicate that Med25 limits adipogenic potential by suppressing the levels of master regulators that govern adipogenesis. Furthermore, we caution the use of early-developmental-stage cardiomyocytes to model adult-stage cells, particularly for dissecting metabolic perturbations emanating from *LMNA* mutations.

## 1. Introduction

The nuclear resident intermediate filament proteins lamin A/C are multi-functional proteins encoded by the *LMNA* gene. Mutations in the *LMNA* gene have been known to cause a wide array of syndromes collectively termed laminopathies, with diseases affecting highly metabolic tissues of mesenchymal origin [1]. As such, striated muscle and adipose tissue diseases, in the form of muscular dystrophy and lipodystrophy, respectively, comprise the vast majority of laminopathy cases [2]. One of these is cardiomyopathy with variable skeletal muscle involvement (herein referred to as *LMNA* cardiomyopathy), which is characterized by dilatation of ventricles due to CM damage and the ensuing pathological remodeling [3]. Current clinical recourse is limited to therapies aiming to mitigate the symptoms of congestive heart failure. Despite considerable efforts, mechanistic underpinnings of how *LMNA* mutations cause disease remain largely elusive, and this lack of basic insights has impeded the development of effective targeted therapies.

Dysregulation of lipid metabolism in cardiomyopathy is a well-established phenomenon, particularly from those arising within the context of obesity [4]. Although originally believed to contribute to disease pathogenesis, it is currently debated whether the observed lipid accumulation in cardiomyopathy occurs as a compensatory response [4]. Nevertheless, end-stage heart failure leads to a deficit of lipid availability/utilization as an energy source, causing the myocardium to rely on glucose and ketones for ATP generation [5]. Notably, myocardial lipid accumulation has been previously described in a knock-in mouse model harboring an *Lmna* p.delK32 mutation [6]. A mutation identified in human patients, the p.delK32 mutation produces lamin A/C proteins with a deletion of a lysine in position 32 in the N-terminal domain, leading to severe cardiac phenotype and early lethality within the first month of life [6].

At the molecular level, a functional link between the nuclear envelope and intranuclear lipids appears to be evolutionarily conserved, and its relevance is just beginning to be elucidated. Intranuclear lipid accumulation has been observed in yeast [7], as well as in mammalian cells [8,9], and plays diverse and essential roles in the nucleus. For example, a series of studies from Hovak and colleagues demonstrated that phosphatidylinositol 4,5-bisphosphate (PIP2) and its binding proteins are abundantly found in the nucleus [10,11]. They subsequently showed that PIP2 acts to recruit and enrich various proteins involved in RNA polymerase II (Pol II)-mediated transcription, creating a compartmentalized space within the nucleus, termed nuclear condensates [12,13]. These studies highlight the novel roles of lipids and their derivatives as well as the importance of maintaining proper lipid metabolism homeostasis necessary for fundamental nuclear processes including transcriptional regulation.

We recently showed in a novel tamoxifen-inducible CM-specific *Lmna* deletion model that fulminant cardiomyopathy and pathological fibrosis develops within 4 weeks post cessation of tamoxifen administration [14]. Coincident with the incipient phase of the disease, in which subtle histological and molecular changes are evident without impacting myocardial pump function, we discovered the upregulation of Med25 protein in a translating mRNA screen [14]. Med25 is a member of the Mediator complex, an evolutionarily conserved multi-subunit complex that integrates cellular signals and relays to the basal Pol II transcriptional machinery to generate an appropriate transcriptional response. Disruption of various mediator subunits has been shown to cause defective cardiac development and cardiomyopathy [15,16,17]. Although Med25 is well characterized in *Arabidopsis thaliana*, particularly regulating the jasmonate signaling pathway [18], comparatively less is known about its role in the mammalian system. Prior studies have linked Med25 to regulating lipid metabolism, nuclear receptors, and ER stress responses [19,20,21]. Interestingly, Med25 has also been demonstrated to bind directly to the polyunsaturated fatty acid arachidonic acid [22], further implicating its function in regulating lipid metabolism/signaling. Interestingly, jasmonate signaling in plants is analogous to the mammalian eicosanoid pathway [23,24], which further supports the direct connection between Med25 and arachidonic acid from which eicosanoids are derived. Collectively, these studies suggest that Med25 is a stress-responsive mediator member regulating lipid signaling-induced transcriptional responses.

In the current manuscript, we show that lipid accumulation occurs in the adult myocardium following induced CM-specific *Lmna* deletion during the incipient phase of the disease. Similar to the phenotype observed in patients with heart failure, this increase is transient and returns to below baseline at end-stage heart failure. This transient lipid accumulation is concomitant with the upregulation of Med25 protein expression. In vitro experimental models to recapitulate in vivo lipid accumulation in CMs depleted of lamin A/C revealed that the circuitry governing lipid metabolism is divergent depending on the developmental stage of the CMs. By leveraging the well-established 3T3-L1 preadipocyte differentiation system, we demonstrate that Med25 maintains a limit on adipogenic potential by suppressing the levels of master regulators that govern adipogenesis.

## 2. Results

### 2.1. Lipid Accumulation in the Myocardium of CM-Specific Lmna Deletion Model of LMNA Cardiomyopathy

**Figure 1:**
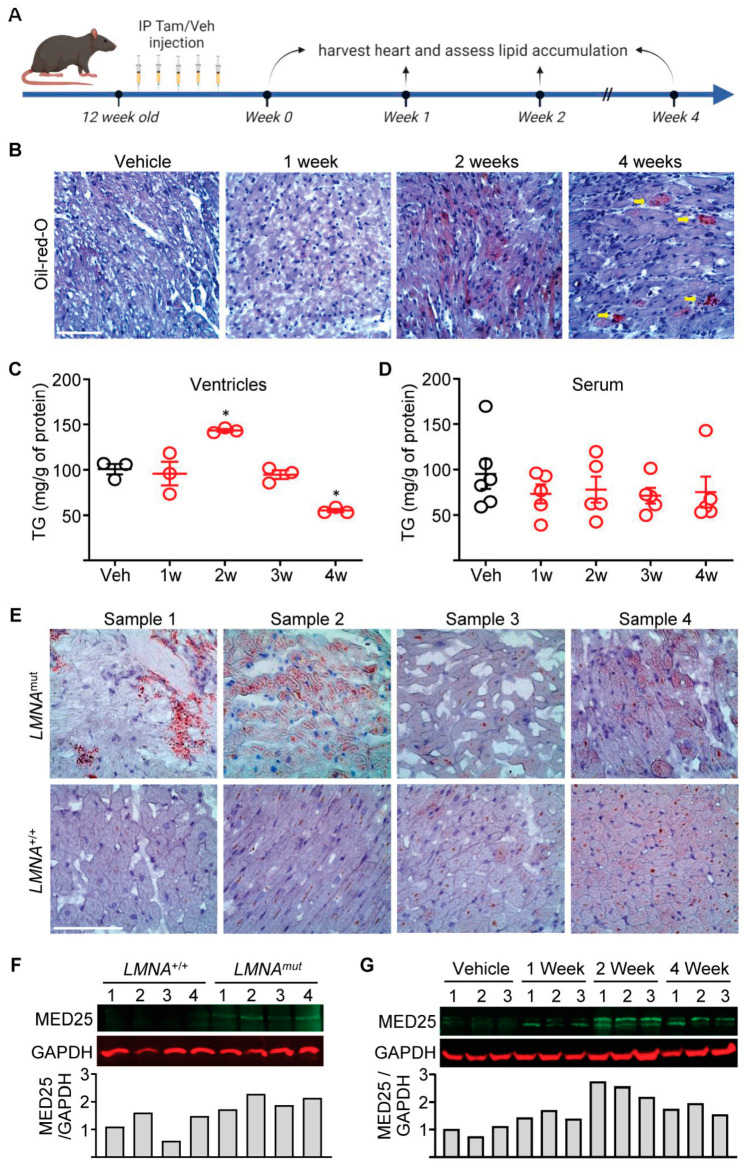
*LMNA* cardiomyopathic hearts accumulate lipids during disease pathogenesis. (**A**) Schematic of tamoxifen (Tam) administration schedule. Syringe denotes days of Tam injection. IP denotes intraperitoneal, Veh denotes vehicle (corn oil). (**B**) Oil-red-O staining of heart sections from CM-CreTRAP: *Lmna*^flox/flox^ mice at 1, 2, and 4 weeks post final Tam dosing. Yellow arrows denote focal lipid accumulation. Representative images from 3 independent sections from 3 mice per group are shown. All scale bars = 100 μm. (**C**) Triglyceride (TG) measurements of the heart tissue extracts from CM-CreTRAP: *Lmna*^flox/flox^ mice treated with vehicle (Veh) or Tam. 1–4w denote weeks post Tam treatment. Error bars denote = SEM. * denotes *p* < 0.005 using one-way ANOVA with Dunnett’s post hoc test. *n* = 3. (**D**) TG measurements of serum from CM-CreTRAP: *Lmna*^flox/flox^ mice treated with vehicle (Veh) or Tam for 1–4 weeks. *n* = 3. (**E**) Oil-red-O staining of human hearts from *LMNA* cardiomyopathy patients (*LMNA*^mut^) and age-/sex-matched non-failing wild-type *LMNA* hearts (*LMNA*^+/+^). Representative images from 10 independent sections per group are shown. (**F**) Immunoblot of MED25 and GAPDH on human hearts as described in (**E**), with quantitation on the bottom. Numbers on top of blots denote individual heart samples. *n* = 4 per group. (**G**) Immunoblot of MED25 and GAPDH on tissue extracts from CM-CreTRAP: *Lmna*^flox/flox^ mice treated with vehicle, 1, 2, and 4 weeks post final Tam dosing. Numbers on top of blots denote individual heart samples. *n* = 3 per group. Quantitation is shown on the bottom.

We recently described a novel cre recombinase driver line referred to as CM-CreTRAP mice [14]. It is a bi-cistronic transgenic line wherein tamoxifen (Tam)-inducible Cre recombinase (CreERT2) and EGFP-L10a fusion protein are co-expressed under the control of a CM-specific promoter, myosin heavy chain 6 (*Myh6*) promoter. EGFP-L10a is a fusion of enhanced green fluorescent protein and ribosomal protein L10a, a component of the 60S ribosomal protein, which allows tagging of polysomes for immunoaffinity purification of translating mRNA termed Translating Ribosome Affinity Purification (TRAP) [25]. These mice develop molecular and histological changes by 2 weeks post Tam administration and a severe decline in cardiac function and pathological fibrosis by 4 weeks [14]. Following 100 mg/kg Tam administration at 12 weeks of age for 5 consecutive days followed by 2 days rest to delete *Lmna* specifically in CMs, we assessed lipid accumulation by oil-red-O staining at the indicated time points (Figure 1A and Supplementary Figure S1). We observed significant CM lipid accumulation at 2 weeks post Tam treatment (Figure S1). By 4 weeks, the lipid accumulation was far less pervasive and more focal, which resembled the pattern of progression in human heart failure [5].

To validate our oil-red-O staining results and to ensure that the lipid accumulation observed in CMs is not due to overall increases in the circulation, we measured triglyceride levels in both the myocardial tissue and blood serum from these mice. Consistent with our histological data, we noted significant lipid accumulation at 2 weeks post Tam that returned to baseline and a deficit at 3 and 4 weeks post Tam, respectively (Figure 1C,D). Despite the observed pattern of lipid accumulation in the myocardial tissue, no significant changes in the triglyceride levels were noted in the circulating serum, demonstrating that (1) *Lmna*-deleted CMs accumulate lipids at 2 weeks post Tam, (2) this increase in accumulation is not due to overall higher lipid levels in the circulation, and (3) the pattern of lipid accumulation in our induced CM-*Lmna* deletion mice recapitulates the phenotype in the human disease of heart failure. To determine whether a similar phenomenon can be observed in the human disease, we performed oil-red-O staining on heart sections from human patients (*LMNA*^mut^) along with age-/sex-matched controls. In three out of four hearts from *LMNA* cardiomyopathy patients, we noted obvious focal lipid accumulation, whereas age-/sex-matched non-failing hearts showed far less and more diffuse staining (Figure 1E).

We previously showed that there is a gradual elevation of Med25 protein expression that reaches its peak by 2 weeks post Tam in hearts of CM-*Lmna*-deleted mice [14]. To determine whether a similar elevation of Med25 protein expression occurs in the human disease, we performed immunoblot analyses on myocardial tissue from human patients and compared them to age-/sex-matched controls (Figure 1F). Despite the inherent variability and the advanced stage of the human disease, we consistently observed higher protein expression of MED25 in the patient samples (Figure 1F). We confirm this in our mouse model, in which MED25 expression remains elevated at 4 weeks post Tam (but lower than the peak at 2 weeks), despite an overall deficit in lipid availability (Figure 1G). Of note, we consistently detected multiple MED25 bands in hearts of CM-*Lmna*-deleted mice. The PhosphoSitePlus^®^ database shows that Med25 can be phosphorylated, ubiquitylated, acetylated, and methylated. Furthermore, there are five transcript variants in mice (but only two variants in humans), in which a putative variant-switching mechanism may also explain the multiple bands. Although the nature of the observed multiple bands is currently unclear, these results indicate that our CM-specific *Lmna*-deletion mouse model recapitulates the human phenotype with regard to lipid accumulation and MED25 expression.

### 2.2. Lmna Deletion Causes Lipid Accumulation in Early Development CMs

**Figure 2:**
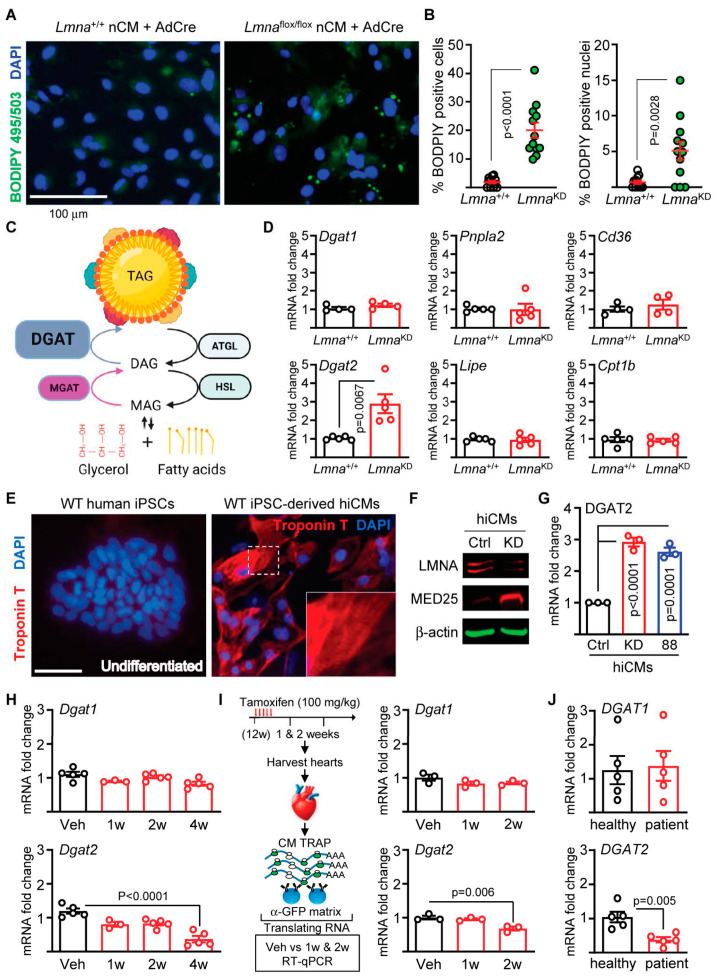
Increased DGAT2 mRNA expression in response to lamin A/C depletion in neonatal and embryonic but not in adult CMs. (**A**) nCMs isolated from *Lmna^+^*^/+^ and *Lmna*^flox/flox^ mice (*Lmna* KD) infected with AdCre and cultured on 50 kPa matrix for 48 h, after which they were stained with BODIPY 495/503 and DAPI. Representative images from *n* = 3 experiments are shown. Scale bar = 100 μm. (**B**) Left panel shows quantitation of BODIPY-positive cells represented as % of BODIPY-positive cells (>1.5 μm diameter) from total number of cells counted. The individual data points denote % of BODIPY-positive cells per image for each group from three independent experiments. Right panel shows % of nuclear BODIPY-positive cells from the same set of experiments. (**C**) Schematic of triglyceride (TAG) metabolism and catabolism with enzymes that catalyze the reaction. DAG and MAG denote diacylglycerol and monoacylglycerol, respectively. TAG is shown encapsulated by a phospholipid monolayer also bound by lipid droplet binding proteins (multi-colored motifs). (**D**) qPCR analyses of *Dgat1*, *Dgat2*, *Pnpla2* (encoding ATGL), *Lipe* (encoding HSL), *Cd36*, and *Cpt1b* on mRNA isolated from *Lmna^+^*^/+^ and *Lmna*^flox/flox^ mice (*Lmna* KD) infected with AdCre. All fold-change values were derived relative to the mean of five *Lmna^+^*^/+^ samples. *n* = 5. *p* values were derived using unpaired, two-tailed Student’s t test. (**E**) Troponin T and DAPI staining on wild-type undifferentiated human iPSCs (left panel) and those differentiated into CMs (hiCMs) (right panel). The dashed white box denotes inset, which shows sarcomeric striations. Scale bar = 100 μm. (**F**) Immunoblot analyses of hiCMs infected with lentivirus carrying either a blank vector (Ctrl) or encoding shRNA targeting *LMNA* (KD) probed for LMNA, MED25, and β-actin. A representative blot is shown from three independent experiments. (**G**) qPCR analyses probing *DGAT2* mRNA expression in Ctrl and KD hiCMs as indicated in 2F as well as hiCMs derived from SCVI88 iPSCs (88). Fold-change values were derived by setting the Ctrl sample value as 1. *p* values were derived using one-way ANOVA with Dunnett’s correction. *n* = 3. (**H**) qPCR analyses of *Dgat1* (top) and *Dgat2* (bottom) mRNA expression in myocardial tissue of CM-CreTRAP: *Lmna*^flox/flox^ mice at 1 to 4 weeks post Tam dosing. Veh denotes vehicle (corn oil). Fold-change was derived from the mean value of 5 Veh samples used as a relative reference. *p* values were derived using one-way ANOVA with Dunnett’s correction. *n* = 5 for all groups except for 1w (*n* = 3). (**I**) Schematic of experimental design using TRAP. Right panel shows qPCR analyses on CM-specific translating mRNAs probed for *Dgat1* and *Dgat2* from CM-CreTRAP: *Lmna*^flox/flox^ mice 1 and 2 weeks post Tam treatment. *p* values were derived using one-way ANOVA with Dunnett’s post hoc. Fold-changes with the mean value from Veh group as a reference control. *n* = 3. (**J**) qPCR analyses of *DGAT1* and *DGAT2* in myocardial tissue from human patients. Fold-change was calculated relative to the mean value from five healthy patient samples. *n* = 5 per group. *p* values were derived using unpaired, two-tailed Student’s *t*-test. All error bars denote SEM in this figure.

Given the lipid accumulation in the *Lmna*-deleted CMs in vivo, we sought to determine whether the depletion of lamin A/C directly causes lipid accumulation. To achieve this, we employed neonatal CMs (nCMs) isolated from *Lmna*^+/+^ and *Lmna*^flox/flox^ mice coupled with adenoviral delivery of cre recombinase (AdCre) to delete *Lmna* in vitro. nCMs depleted of lamin A/C by this method recapitulate many in vivo phenotypes, including MED25 upregulation, observed in the hearts of CM-specific *Lmna*-deleted mice [14], suggesting that this may be a viable model to study CM lipid accumulation in response to lamin A/C depletion. The AdCre-treated cells were then stained with BODIPY 495/503, which stains neutral lipids. AdCre treatment of nCMs isolated from *Lmna*^flox/flox^ resulted in increased presence of lipid droplets but not in nCMs from *Lmna*^+/+^ (Figure 2A). Our quantitative assessment revealed that an average of ~20% of lamin A/C-depleted nCMs contained lipid droplets (Figure 2B, left). We also observed increased intranuclear lipid accumulation in the *Lmna*^KD^ nCMs relative to the *Lmna*^+/+^ counterparts (Figure 2B right and Supplementary Figure S2A). Despite their presence, only a small subset of nuclei (~5%) contained visible lipid droplets, so their significance is currently unknown. These results indicate that the deletion of the *Lmna* gene, and the resulting depletion of lamin A/C proteins, directly elicits lipid accumulation.

To begin to pinpoint the underlying mechanisms of lipid accumulation following lamin A/C depletion, we initially focused on the enzymatic pathways regulating triglyceride generation (*Dgat1* and *Dgat2*) and breakdown (*Pnpla2* encoding ATGL and *Lipe* encoding HSL) as shown in the schematic in Figure 2C. Following *Lmna* deletion in nCMs, we performed RT-qPCR analyses to ascertain the levels of mRNA transcripts encoding the genes indicated above (Figure 2D). We observed that *Dgat2* is specifically elevated in response to lamin A/C depletion, which is consistent with increased lipid accumulation (Figure 2D). Additionally, we also assessed fatty acid transporters (*Cd36* and *Cpt1b*) and observed no significant changes with lamin A/C depletion (Figure 2D). 

We then assessed the generalizability of elevated *Dgat2* in response to lamin A/C depletion. To achieve this, we utilized human induced CMs (hiCMs) derived from induced pluripotent stem cell (iPSC) line SCVI114 [26], which is the wild type for the *LMNA* gene. Following differentiation into CMs using a previously established procedure [27] (Supplementary Figure S2A), we observed spontaneously contracting CMs (Supplementary Figure S2B and associated video file) as well as robust expression of CM-specific markers such as *Tnnt2* and *Myh6* (Supplementary Figure S2C). Furthermore, immunofluorescence analysis using anti-troponin T antibodies revealed not only a positive staining, but the expressed troponin T proteins spontaneously arranged themselves into sarcomere-like striations (Figure 2E).

We then depleted lamin A/C in the hiCMs using short hairpin-mediated silencing via lentiviral vectors. Having confirmed knockdown of lamin A/C (Figure 2F), we measured *DGAT2* transcripts and observed a ~3-fold elevation in hiCMs with lamin A/C depletion compared to controls (hiCMs infected with lentiviruses carrying a blank shRNA) (Figure 2G). To ascertain whether a similar elevation of *DGAT2* transcripts is induced in response to the expression of a mutant variant of lamin A/C (instead of a deletion model), we derived hiCMs from an IPSC line SCVI88 [26] that was generated from a patient identified with a K117fs mutation [28]. hiCMs derived from this iPSC line also displayed elevated DGAT2 transcripts relative to hiCMs from SCVI114 (Figure 2G), despite lacking an isogenic control for an ideal comparison. Nevertheless, our data indicate that both human and mouse CMs with disrupted *LMNA* gene expression display increased expression of *DGAT2*.

### 2.3. Distinct Mechanisms Underlying Lipid Regulation between Adult and Early Development CMs

Having shown that CMs depleted of lamin A/C elicit *Dgat2* expression, we sought to determine whether similar increases are observed in the myocardium of our CM-specific *Lmna*-deletion mice. We isolated mRNA from ventricular tissue after 1–4 weeks post Tam dosing and performed RT-qPCR to measure *Dgat1* and *Dgat2* transcript levels. Compared to vehicle (corn oil) controls, we observed no elevation of *Dgat1* and *Dgat2* levels (Figure 2H). To the contrary, *Dgat2* levels were significantly reduced at the 4 week time point relative to the vehicle control (Figure 2H, bottom panel). Given that the myocardial tissue is heterocellular in nature, we aimed to remove the contribution from other cell types in the heart. We achieved this by performing TRAP on the hearts isolated from mice treated with Tam to immunopurify translating mRNA specifically from CMs (Figure 2I). No elevation of *Dgat1* and *Dgat2* expression was observed (Figure 2I, right panel); their expression pattern resembled those observed from the ventricular tissue, further indicating that *Dgat2* is not elevated in the hearts of adult mice with CM-specific *Lmna* deletion. Finally, we measured *DGAT1* and *DGAT2* in myocardial tissue from human patients as well as their sex-/age-matched controls and observed no differences in *DGAT1* but a significant decrease in *DGAT2* (Figure 2J), which again is consistent with our observations in hearts from the CM-specific *Lmna*-deletion mice. Taken together, our results indicate that lipid metabolism perturbations emanating from lamin A/C depletion may be mutually exclusive depending on the developmental state of the CMs. Hence, we caution the use of CMs of neonatal and embryonic origin, while much more amenable for experimental manipulation, as a model to dissect mechanisms underlying metabolic perturbations of adult CMs arising from *LMNA* mutations.

### 2.4. Med25 Depletion Enhances Adipogenesis

**Figure 3:**
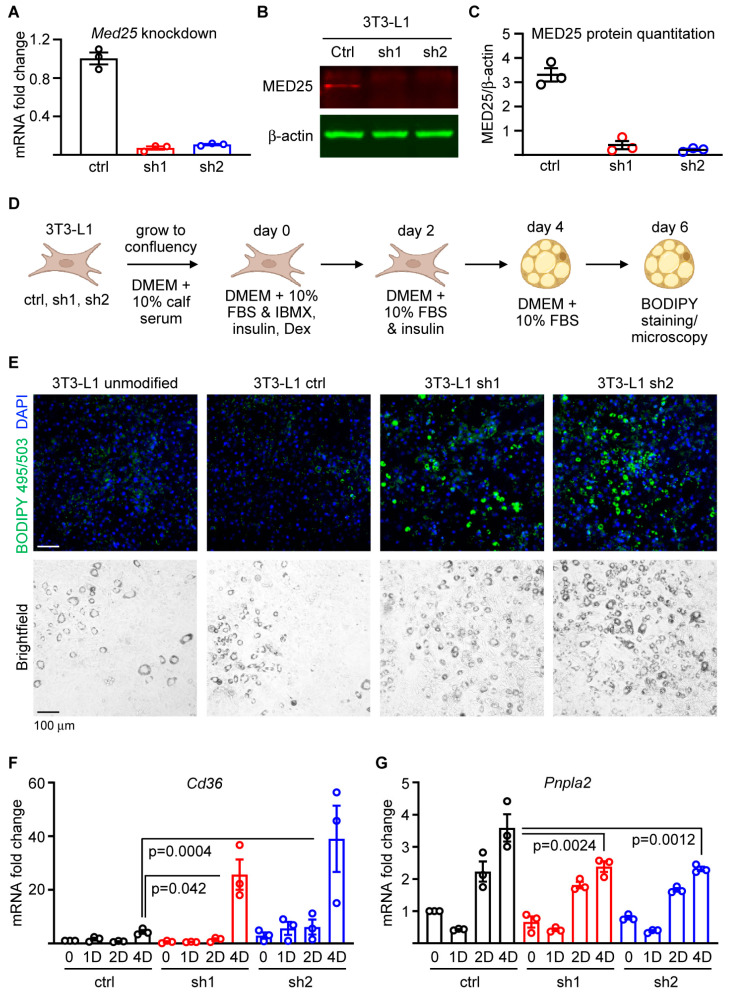
Med25 silencing enhances adipogenesis. (**A**) mRNA expression analysis validating *Med25* expression knockdown relative to controls (ctrl–3T3-L1 infected with lentiviruses carrying blank vector) using two independent shRNAs (sh1 and sh2) that target *Med25*. Fold-change was derived from the mean value of ctrl samples used as a relative reference. *n* = 3. Error bars = SEM. (**B**) Immunoblot of MED25 and β-actin in nuclear extracts from 3T3-L1 cells expressing sh1 and sh2. (**C**) Densitometry measurements of MED25 protein levels normalized to β-actin and represented in arbitrary units. *n* = 3. Error bars = SEM. (**D**) Adipocyte differentiation schema using the ctrl and two (sh1, sh2) Med25 KD cells. Dex denotes dexamethasone. (**E**) BODIPY 495/503 and DAPI staining on 3T3-L1 cells differentiated into adipocytes for 6 days. Bright-field image is also shown to reveal lipid-filled, differentiated adipocytes. Representative images from three independent differentiation experiments are shown. Scale bar = 100 μm. (**F**,**G**) qPCR analyses of *Cd36* (**F**) and *Pnpla2* (**G**) at undifferentiated (0), 1 day (1D), 2 days (2D), and 4 days (4D) post initiation of adipogenic differentiation. *p* values were derived using one-way ANOVA with Tukey’s post hoc. Fold-change values were derived by setting the ctrl 0 h sample value for each respective set as “1”. *n* = 3. Error bars = SEM.

Given the divergent *Dgat2* expression profiles between adult and early-development CMs, we pursued a different strategy to determine the functional relevance of Med25 in lipid accumulation. We employed the well-established 3T3-L1 preadipocyte model, in which we depleted Med25 by shRNA-mediated silencing, followed by differentiation induction into adipocytes by dexamethasone, IBMX, and insulin. We employed two previously described shRNAs [14] to deplete Med25 in 3T3-L1 cells and show that both shRNAs are able to achieve ~90% knockdown (KD) of *Med25* mRNA (Figure 3A) and ~90% and ~95% KD at the protein levels for sh1 and sh2, respectively, relative to 3T3-L1 cells expressing blank shRNA controls (Figure 3B,C).

Following confirmation of Med25 KD, we induced these cells to differentiate into adipocytes according to the schematic in Figure 3D. We also included unmodified 3T3-L1 cells to ensure that the lentiviral transduction in and of itself does not alter the differentiation phenotype. At day 6 post initiation of differentiation, we stained the cells with BODIPY and observed that 3T3-L1 cells depleted of Med25 (with either sh1 or sh2) displayed enhanced adipocyte differentiation as reflected by lipid accumulation (Figure 3D,E; full-well micrographs are shown in Supplementary Figure S3). No observable differences were noted between unmodified 3T3-L1 cells and those infected with lentiviruses carrying blank shRNA, indicating that the lentiviral infection itself did not affect the differentiation efficiency (Figure 3D,E and Supplementary Figure S3). We then assessed transcript levels of various adipocyte markers. During our qPCR analyses, we noted that the typically used internal control genes such as *Gapdh*, *Actb*, and *Hprt* all displayed a pattern of expression that varied by more than 1 CT (cycle threshold) value (Supplementary Figure S4). We tested additional genes as potential candidates (*Rpl13a*, *B2m*, and *Mapk1*) for use as internal controls and found that *Mapk1*, which encodes ERK2 (p42), displayed the lowest CT value variance from undifferentiated 3T3-L1 to 4 days post adipogenic differentiation (Supplementary Figure S4). Based on the foregoing, all qPCR data relating to adipogenic differentiation were normalized to *Mapk1* mRNA values. Our assessment of various adipocyte markers revealed that *Cd36* and *Pnpla2* levels were significantly elevated on day 4 in cells with Med25 KD relative to control cells. This observation further supports the notion that the depletion of Med25 enhances adipocyte differentiation and lipid accumulation. Based on these results, we conclude that Med25 expression acts to dampen adipogenesis and lipid accumulation.

### 2.5. Med25 Depletion Elevates the Expression of Adipogenic Master Regulators

In order to determine the underlying mechanism responsible for the enhanced adipogenesis mediated by Med25 KD, we dissected the signaling pathways and transcriptional regulators that govern differentiation into adipocytes. Molecular regulation governing adipogenesis in 3T3-L1 preadipocytes is complex and involves successive waves of primary, secondary, and even tertiary transcriptional regulators in an adipogenic transcriptional cascade. We focused our analyses on three major areas in the adipogenic cascade: (1) core kinase phosphorylation cascades important for establishing early-stage differentiation such as ERK1/2 as well as AKT signal transduction pathways, (2) cell-type-agnostic transcription factors involved in adipogenesis including the CCAAT-enhancer-binding protein (C/EBP) family of transcription factors [29,30,31], and (3) cell-type-restricted master regulators such as the peroxisome proliferator-activated receptor (PPAR) family of nuclear hormone receptors, some of which are regulated by C/EBP proteins [29,31].

We initiated our studies assessing ERK1/2 (Thr202/Tyr204) and AKT (Ser473) phosphorylation status during the early phase (15 min and 3 h) as well as throughout the rest of the differentiation process (1, 2, 4 days). We observed that the patterns of both total and phosphorylated ERK1/2 between control and Med25 KD cells were virtually identical (Supplementary Figure S5A). Interestingly, despite little to no change in the *Mapk1* mRNA expression as shown earlier, the encoded protein (p42/ERK2) expression increased during the latter part of the differentiation (Supplementary Figure S5A), suggesting that these changes are driven at the post-transcriptional level. Moreover, the levels of total and phosphorylated AKT were also comparable between control 3T3-L1 and the two Med25 KD cells (Supplementary Figure S5B). These results indicate that the core signal transduction pathways such as ERK and AKT cascades are not impacted by the depletion of Med25.

**Figure 4:**
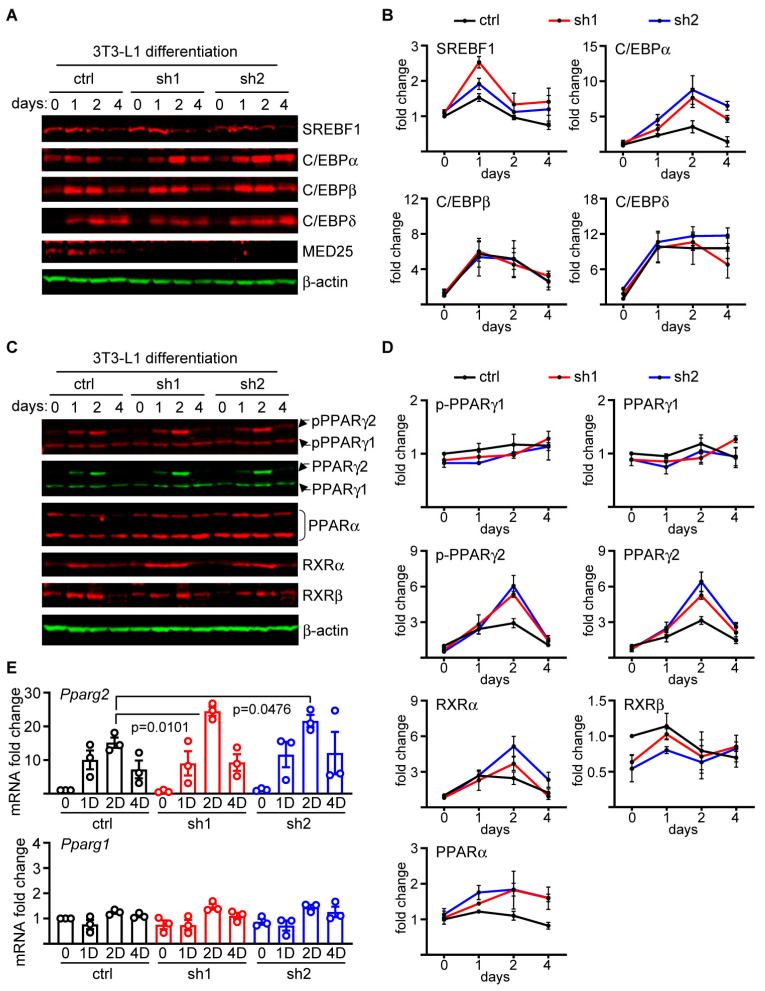
Med25 suppresses the expression of adipogenic master regulators. (**A**) Kinetics of protein expression analyses on control and MED25-depleted 3T3-L1 cells (sh1 and sh2) and differentiated into adipocytes probed for SREBF1, C/EBPα, C/EBPβ, C/EBPδ, MED25, and β-actin (loading control). Cells for protein extraction were collected on days 0 (undifferentiated), 1, 2, and 4 after differentiation initiation as shown in Figure 3D. Representative blots are shown out of *n* = 3 experiments. (**B**) Quantitation of the immunoblot analyses shown in 4A. The densitometry values for the probed proteins were normalized to β-actin and represented as fold-change relative to 0 hr, which was set as “1”. Error bars denote SEM from three independent experiments. (**C**) Kinetics of protein expression analyses on MED25-depleted 3T3-L1 cells differentiated into adipocytes as in 4A but probed for phospho-Ser273 PPARγ1 (pPPARγ1) and pPPARγ2, total PPARγ1 and PPARγ2, PPARα, RXRα, RXRβ, and β-actin. Representative blots are shown out of *n* = 3 experiments. (**D**) Quantitation of the immunoblot analyses shown in 4C. The densitometry values for the probed proteins were normalized to β-actin and represented as fold-change relative to 0 h, which was set as “1”. Error bars denote SEM from three independent experiments. (**E**) qPCR analyses of *Pparg1* and *Pparg2* mRNA expression at undifferentiated (0), 1 day (1D), 2 day (2D), and 4 day (4D) post initiation of adipogenic differentiation. *p* values were derived using one-way ANOVA with Tukey’s post hoc. Fold-change values were derived by setting the ctrl “0” sample value for each respective sets as “1”. *n* = 3. Error bars = SEM.

We then assessed transcription factors involved in adipogenesis that are expressed in various tissues. We focused on Sterol Regulatory Element-Binding Transcription Factor 1 (SREBF1) and C/EBP family members, as they are well established to regulate the activity of adipogenic master regulator PPARγ [29,31,32]. We observed peak SREBF1 expression on day 1 of the differentiation process, and although the Med25 KD cells appeared to express higher levels than control cells during the peak expression, the extent of the increase was variable (Figure 4A,B). However, the largest difference in expression was observed in C/EBPα; we noted peak expression on day 2 that was significantly enhanced in cells with the Med25 KD (Figure 4A,B). Moreover, whereas C/EBPα returned to baseline by day 4 in control 3T3-L1, it remained elevated in cells with Med25 KD, further validating the enhanced C/EBPα phenotype (Figure 4A,B). Notably, the elevated C/EBPα expression occurred despite the comparable levels of its activators C/EBPβ and C/EBP expression (Figure 4A,B). As expected, we did not detect Med25 in the KD cells, but interestingly, its expression in control cells decreased with adipogenic differentiation, further supporting the notion that Med25 expression antagonizes adipogenesis (Figure 4A).

C/EBPα plays an important role in regulating the expression level of PPARγ [29,31], a master regulator of adipogenesis. Given that Med25 KD enhances C/EBPα expression in the context of adipogenesis, we reasoned that there would be similar increases in the PPARγ activity and/or expression. There are two PPARγ isoforms generated by differential splicing and promoter usage [33]: PPARγ1, which is expressed in other cell types, and PPARγ2, which is largely restricted to adipocytes [33]. Serine 273 phosphorylation of PPARγ, which was shown to play a critical role in obesity and insulin resistance, is mediated by the Cdk5/ERK1/2 axis [34]. Therefore, we measured the Ser273 phosphorylation status as well as the expression levels of PPARγ1 and PPARγ2. Our immunoblot analyses revealed that although no obvious differences were noted for both the total and phospho-PPARγ1 (pPPARγ1) levels between the Med25 KD and control 3T3-L1 cells during adipocyte differentiation, we observed significant increases for PPARγ2 in Med25 KD cells (Figure 4C,D). The expression patterns between pPPARγ2 and total PPARγ2 were virtually identical, indicating that the rate-limiting step for Ser273 phosphorylation of PPARγ2 is its expression. PPARα, which can also contribute to adipogenesis, was similarly elevated in the Med25 KD cells on day 4 relative to controls, but the extent of the increase was modest, which further suggests that the enhanced adipogenicity is driven primarily by de novo PPARγ2 expression (Figure 4C,D). We also measured the levels of retinoid-X receptor isoforms as they form heterodimers with PPARγ and utilize Med25 to mediate their transcriptional activation potential. We observed no significant differences in RXRα and RXRβ between cells with and without Med25, indicating that no compensatory mechanisms affect these nuclear receptors (Figure 4C,D). RXRγ is a muscle-restricted isoform, and we did not detect its expression in our control or Med25 KD 3T3-L1 cells (data not shown). Finally, we show that the kinetics of the increased expression of PPARγ2 were consistent with a similar pattern of increase in the *Pparg2* mRNA expression (Figure 4E), indicating that the observed effect is mediated at the level of transcription. No increases were noted for *Pparg1*, which is consistent with the observed protein expression for PPARγ1 (Figure 4E). Taken together, we conclude that Med25 maintains a limit on adipogenic potential by suppressing the levels of C/EBPα and PPARγ2 that govern adipogenesis.

## 3. Discussion

In this study, we explored the functional relevance of Med25 in lipid accumulation during adipogenesis and show that depletion of Med25 increased lipid accumulation during 3T3-L1 differentiation into adipocytes. Furthermore, our data indicate that the increased lipid accumulation is due to enhanced differentiation into adipocytes, driven primarily by the increased expression of adipogenic master regulators PPARγ2 and C/EBPα. Although we observed an elevation of PPARα in response to Med25 silencing as well, it was relatively modest compared to PPARγ and C/EBPα. 

The impetus of our studies was based on our observation that Med25 was elevated at the protein level following CM-specific *Lmna* deletion in vivo prior to any functional decline in cardiac performance [14]. Its increased expression coincided with diffuse and pervasive myocardial lipid accumulation, suggesting a functional link between the emergence of Med25 and myocardial lipid accumulation. This is further supported by a previously reported study demonstrating Med25 as a lipid-binding protein [22]. Based on our results using 3T3-L1 preadipocytes as a model, it is attractive to hypothesize that the expression of Med25 in response to *Lmna* deletion in CMs underlies the clearance of lipids with the worsening disease, but how this is achieved remains unclear. Med25 was previously shown to regulate lipid metabolism in the liver by acting as a coactivator for liver-specific transcription factor HNF4α [20], which is not expressed in CMs. Moreover, our current study demonstrates that Med25 depletion enhances the expression of PPARγ2, which is restricted to adipose tissue [35]. Therefore, a similar molecular circuitry, but with different set of specific “actors”, likely governs the lipid dysregulation observed in the hearts with CM-specific *Lmna* deletion.

Our observation showing lipid droplet accumulation, particularly inside the nucleus, in response to lamin A/C warrants further investigation. Lipid accumulation is a hallmark of endoplasmic reticulum (ER) stress, and given that the nuclear envelope is contiguous with the ER membrane, the depletion of lamin A/C likely causes ER stress. Consistent with this notion, we observed a selective activation of the ER stress response following *Lmna* deletion in vivo [14]. Furthermore, the ER, as well as the nuclear envelope, has been shown to be a site of lipid droplet formation [7,8], further implicating lamin A/C with lipid accumulation. As more nuclear-resident processes that depend on lipids are discovered (e.g., PIP2 regulation of nuclear condensates [12,13]), they will help explain why a cell under stress would commit to an energy-intensive process of generating neutral lipids.

We established culture models of CMs with in vitro lamin A/C-depletion to dissect the underlying mechanisms. Although lipid droplet formation is induced in response to *Lmna* deletion in nCMs, our data presented here demonstrate that the metabolic circuitry in response to lamin A/C-depletion in early developmental CMs (nCM and hiCMs) is different from adult CMs. This is not surprising, given the multitude of distinct characteristics that define nCM and adult CMs, from simple morphological differences (that impact mitochondrial distribution) [36], to the ability to regenerate [37], as well as fuel-type switching from reliance on glycolysis to oxidative phosphorylation [38]. Therefore, extreme caution should be taken when using nCMs and hiCMs to model adult CM phenomena; concordance in biochemical and molecular phenotypes should be confirmed between the developmentally distinct CMs prior to extrapolating data between them.

## 4. Materials and Methods

### 4.1. Animals

All animal procedures were approved by the Institutional Animal Care and Use Committee of Thomas Jefferson University. All methods adhered to the NIH Guide for the Care and Use of Laboratory Animals. *Lmna*^flox/flox^ mice [39], procured from The Jackson Laboratory on a mixed background, were backcrossed to C57BL/6J mice for a minimum of 8 generations and genotyped as indicated by the distributor. CM-CreTRAP transgenic mice were generated in a C57BL/6 background by Cyagen Biosciences. The construction of the bi-cistronic transgene was described in detail in [14]. Genotyping was performed on genomic DNA purified from tail clippings by standard PCR using primers indicated in Supplementary Table S2. The mice were housed in a disease-free barrier facility with 12/12 h light/dark cycles and fed a chow diet ad libitum. Tamoxifen (MilliporeSigma, St. Louis, MO, USA, cat# T5648) was reconstituted in corn oil (MilliporeSigma, St. Louis, MO, USA, cat# C8267) and delivered intraperitoneally. 

### 4.2. Human Samples

Heart tissue from human subjects with *LMNA* cardiomyopathy were obtained at the time of heart transplantation. Sex- and age-matched control myocardium was obtained from brain-dead organ donors. Use of human heart tissue for research was approved by the University of Pennsylvania Institutional Review Board, and the use of hearts from brain-dead organ donors for was approved by the Gift-of-Life Donor Program in Philadelphia, PA, USA. Detailed methods for harvesting of tissue can be found in [14]. The human samples were obtained through a Uniform Biological Materials Transfer Agreement with The Trustees of the University of Pennsylvania. The samples were collected de-identified and not specifically for the proposed research by interacting with living individuals.

### 4.3. Primary nCM Isolation and Lmna Deletion

Primary murine nCMs were isolated from the ventricles of 1–2 day old wild-type C57BL/6 and *Lmna*^flox/flox^ mouse pups using MACS neonatal heart dissociation kit according to the manufacturer (Miltenyi Biotec, Bergisch Gladbach Germany cat# 130-098-373) as previously described [14]. Following isolation, nCM cells were plated onto 10 μg/mL laminin-coated wells with 50 kPa hydrogels (Matrigen, Irvine, CA, USA, cat# SW12-EC-50 PK). To prevent the potential growth of non-myocyte cells, the culture media was also supplemented with 100 μM 5-bromo-2-deoxyuridine (BrdU) and 10 µM cytosine arabinoside (Ara-C). The cells were maintained at 37 °C in 95% humidity with 5% CO_2_ concentration. The cells were allowed to attach overnight, after which the medium (composed of DMEM + 10% FBS + 10 µM Ara-C) was replaced after 2x rinse with PBS to remove dead cells. To delete *Lmna* in vitro, adenovirus carrying mCherry-Cre (AdCre) (Vector Biolabs, Malvern, PA, USA, cat# 1773) was used at 50 MOI. Comparable infection efficiencies were confirmed by mCherry.

### 4.4. iPSC-Derived hiCMs

Human iPSC lines were obtained from Dr. Joseph C. Wu at the Stanford Cardiovascular Institute. They were derived by reprogramming PBMC of a 41-year-old healthy Asian male volunteer (SCVI-114) and human dermal fibroblast of a 60-year-old Asian male patient having the K117fs *LMNA* mutation (SCVI-88) [28]. The iPSC lines were cultured and differentiated into hiCMs according to a previously published protocol [27]. Contracting organoids emerged around day 7 of differentiation with maximal contraction achieved by 15–21 days. The beating organoids were kept in culture for 4 weeks before dissociating to single-cell suspension for metabolic selection of hiCMs [40]. To dissociate the beating organoids into single-cell suspension, they were trypsinized with 0.05% trypsin for 10–15 min at 37°. After centrifugation at 800–900 rpm for 5 min, the cells were seeded in matrigel-coated plates at high confluency with DMEM media with 5% FBS, bFGF2 (ProspecBio, East Brunswick, NJ, USA, cat# cyt-557-b), and ROCK inhibitor (Y-27632; Selleck Chemicals, Houston, TX, USA, cat# S1049). The next day, the medium was changed to glucose- and pyruvate-free DMEM (ThermoFisher Scientific, Waltham, MA, USA, cat# 11966025) but supplemented with 5% FBS, 4 mM lactate (MilliporeSigma, St. Louis, MO, USA, cat# L7022), and b-FGF2. Cells were replenished with fresh media every 2 days with vigorous washing to get rid of dead cells of non-CM population. 

### 4.5. 3T3-L1 Culture, shRNA Knockdown, and Adipogenic Differentiation

3T3-L1 cells (American Type Culture Collection, Manassas, VA, USA, cat# CL-173) were maintained in DMEM supplemented with 10% FBS at 37 °C with 5% CO_2_ and subcultured at ~60–70% confluency. Viral packaging cell line 293T cells were maintained in the same media. For stable knockdown of *Med25*, we used two independent shRNAs in the pLKO.1 lentiviral vector backbone identified from murine *Med25* shRNA (MilliporeSigma, St. Louis, MO, USA, cat# SHCLNG-NM_029365) with the sequences GCAGCTGTTCGATGACTTTAA (shRNA1) and TGCAGCTGTTCGATGACTTTA (shRNA2). For stable knockdown of *LMNA* in hiCMs, shRNA with the following sequence was used: AAGCAACTTCAGGATGAGATC. The lentiviral vectors were co-transfected into 293T cells with the packaging vectors pCMV-dR8.2 dvpr and pCMV-VSV-G (cat# 8455 and 8454, respectively, from Addgene, Watertown, MA, USA). Virus-infected cells were selected with 2 μg/mL puromycin. Adipocyte differentiation was performed according to the protocol available at ATCC with slight modifications. Briefly, the viral-infected 3T3-L1 cells were plated in DMEM with 10% BCS and grown to confluency. Once the cells were fully confluent, control (undifferentiated) samples of each cell type were harvested for RNA/protein, and the remaining samples were treated with MDI induction medium composed of DMEM with 10% FBS, 200 nM insulin (MilliporeSigma, St. Louis, MO, USA, cat# I5500), 11.5 μg/mL IBMX (MilliporeSigma, St. Louis, MO, USA, cat# I7018), and 1 μM dexamethasone (MilliporeSigma, St. Louis, MO, USA, cat# D4902). After 2 days of treatment with the MDI induction medium, the medium was replaced with insulin medium (DMEM with 10% FBS and 200 nM insulin). After 2 days, the medium was replaced with DMEM with 10% FBS.

### 4.6. Protein Extraction, Immunoblot Analysis, and TG Analysis

Samples were homogenized in chilled radioimmunoprecipitation assay (RIPA) buffer (MilliporeSigma, St. Louis, MO, USA, cat# R0278) with Pierce protease-inhibitor cocktail (ThermoFisher Scientific, Waltham MA, USA cat# A32963) and 1 mM sodium vanadate (MilliporeSigma, St. Louis, MO, USA, cat# S6508). After brief sonication (Dismembrator Model F60, ThermoFisher Scientific, Waltham, MA, USA), the samples were prepped in Laemmli buffer, after which 15 to 30 µg of the protein extracts were loaded for SDS-PAGE. Antibodies and the dilutions used in the study are provided in Supplementary Table S1. Proper loading was confirmed by probing with GAPDH antibodies for primary heart tissue and β-actin for cell lines. Immunoblot images were captured using an Odyssey^®^ Fc Imaging System, and densitometry of blots was performed using Image Studio software version 5.2 (LI-COR Biosciences, Lincoln, NE, USA) normalized to loading controls. Uncropped blot images are provided in Supplementary Figure S6. TG content of myocardial tissue and serum was assessed using a Triglyceride Colorimetric Assay Kit (Cayman Chemical, Ann Arbor, MI, USA, cat# 10010303) according to the manufacturer’s instructions.

### 4.7. RNA Isolation, Translating Ribosome Affinity Purification, and RT-qPCR

Total RNA was isolated using a Direct-zol RNA kit (Zymo Research, Irvine, CA, USA, cat# R2053) with a minor modification. Samples were harvested in TRIzol (Zymo Research, Irvine, CA, USA, cat# R2050-1-200), and the aqueous phase containing total RNA was separated by adding chloroform (20% volume of TRIzol). The aqueous fraction was carefully collected, to which 100% molecular grade ethanol was added at a 1:1 ratio and then further processed using the Direct-zol RNA kit according to the manufacturer’s instructions. cDNA was generated from 500 ng of RNA and primed with a 1:1 ratio of random hexameric primers and oligodT using a RevertAid RT kit (ThermoFisher Scientific, Waltham, MA, USA, cat# K1691). qPCR was performed in duplicates with QuantStudio5 qPCR system (Life Technologies) using PowerUP SYBR-green (ThermoFisher Scientific, Waltham, MA, USA, cat# A25743). For data presented in Figure 2, *Gapdh* was assessed to ensure fidelity of enzymatic reactions and used as an internal control to normalize qPCR results. For adipocyte differentiation with 3T3-L1, *Mapk1* was used. Fold-changes in gene expression were determined by the ΔCt method [41] and presented as fold-change (FC) over negative controls. We employed two approaches to generate ΔΔCT: (1) for all qPCR data from tissue samples, the FC of all samples (including controls) was calculated relative to the mean value of control samples. For FC data from cell lines in kinetics experiments (Figure 3F,G and Figure 4E), the value of the control sample at the 0 timepoint was set to “1” and all other samples’ FC calculated relative to this control sample. For TRAP, we processed the ventricular tissue exactly as described previously [25]. Mice (12 weeks old) were treated with either vehicle or Tam as described in Figure 2I. Two weeks after Tam (or vehicle) dosing, ventricular tissue were harvested and translating mRNAs purified from *n* = 3 biologically independent samples. For cDNA synthesis of TRAP mRNA, 100 ng of translating mRNA was used. A complete list of primer sets used in the study is provided in Supplementary Table S2.

### 4.8. Microscopy and Histopathological Analysis

Oil-red-O staining was performed by Translational Research & Pathology Shared Resources (Thomas Jefferson University, Philadelphia, PA, USA) using standard methods. For immunofluorescence, cells were fixed in ice-cold methanol:acetone (3:1) and processed using standard methods with antibodies at listed concentrations in Supplementary Table S2. Cellular neutral lipid staining was performed on paraformaldehyde-fixed nCMs by incubating 10 μM BODIPY 493/503 (ThermoFisher Scientific, Waltham, MA, USA, cat# D3922) dissolved in PBS for 20 min at 37 °C. DAPI was used as a counterstain. Micrographs were captured using an EVOS M7000 Imaging System (ThermoFisher Scientific, Waltham, MA, USA). All image analysis was performed using ImageJ 2.0 software [42]. Quantification of % nCMs containing lipid droplets was performed by dividing the number of cells containing circular BODIPY positive signals larger than 1.5 μm in diameter by the total number of cells (~280 cells per condition) across 6 images per condition from 2 independent experiments.

### 4.9. Statistical Analysis

Statistical analyses were performed using Graphpad Prism 9 (GraphPad Software, Boston, MA, USA). Statistical significance of binary comparisons was determined by a 2-tailed, unpaired Student’s *t*-test, with a value of *p* < 0.05 considered significant. Statistical significance of three or more variables was determined by one-way ANOVA with post-hoc Tukey error correction (for multiple comparisons) or Dunnett’s for comparison to a specific control. *p* < 0.05 was considered significant. Values with error bars shown in figures are means ± SEM unless indicated otherwise. Sample sizes are indicated in the figure legends. 

## Data Availability

All data associated with this study are available in the main text or the Supplementary Materials. Human cardiac samples were obtained through MTA with The Trustees of the University of Pennsylvania. TRAP GFP antibodies were obtained through MTA with Bi-Institutional Antibody and Bioresource Core Facility.

## Supplementary Materials

The following supporting information can be downloaded at: https://www.mdpi.com/article/10.3390/ijms24076155/s1.
