# Supplementary material for: Med25 Limits Master Regulators That Govern Adipogenesis
Source: Int J Mol Sci. 2023 Mar 24;24(7):6155. doi: 10.3390/ijms24076155 (PMC10093881; doi:10.3390/ijms24076155)
Supplement: Supplementary file 1 [file ijms-24-06155-s001.zip › ijms-2265094 Supplementary Figures and Tables.pdf]

Supplementary Figure S1

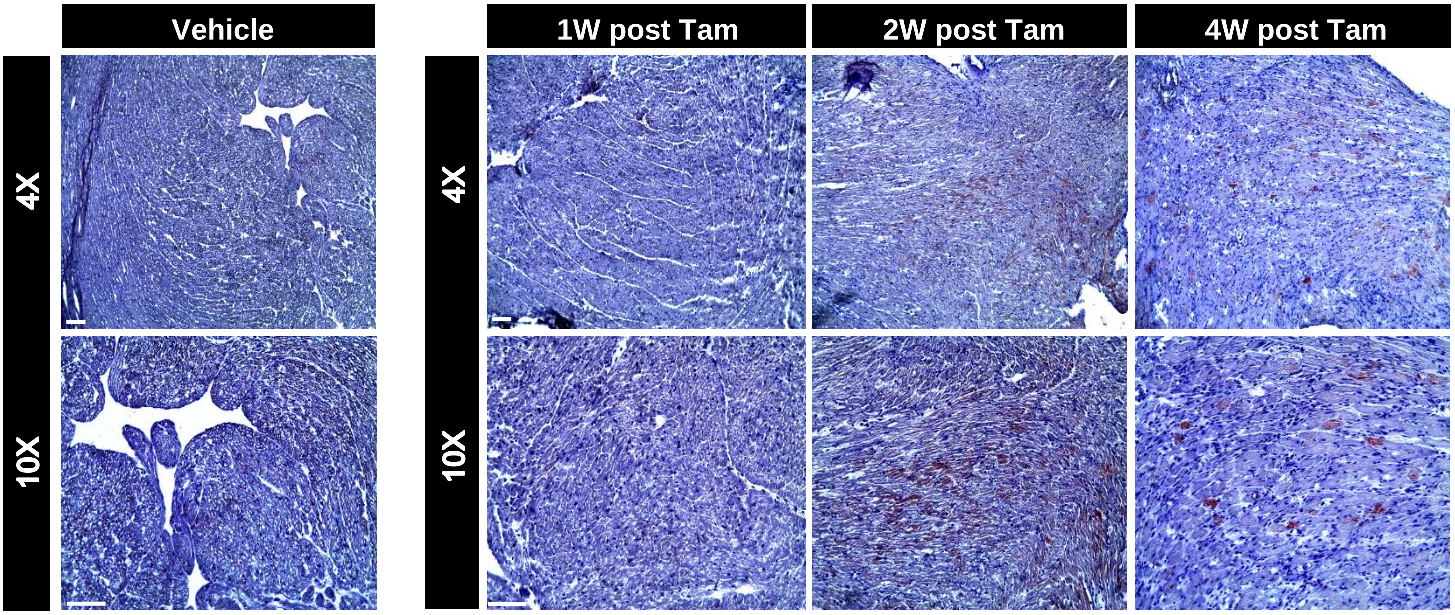

Figure S1. Lower magnification images of Oil-red-O staining for Figure 1B. 10 and 4x images of the heart sections from CM-CreTRAP:*Lmna*$^{flox/flox}$ mice at 1, 2, and 4 weeks post final Tam dosing are shown. Vehicle treated heart images are shown on the left. Scale bars = 100 μm.

Supplementary Figure S2

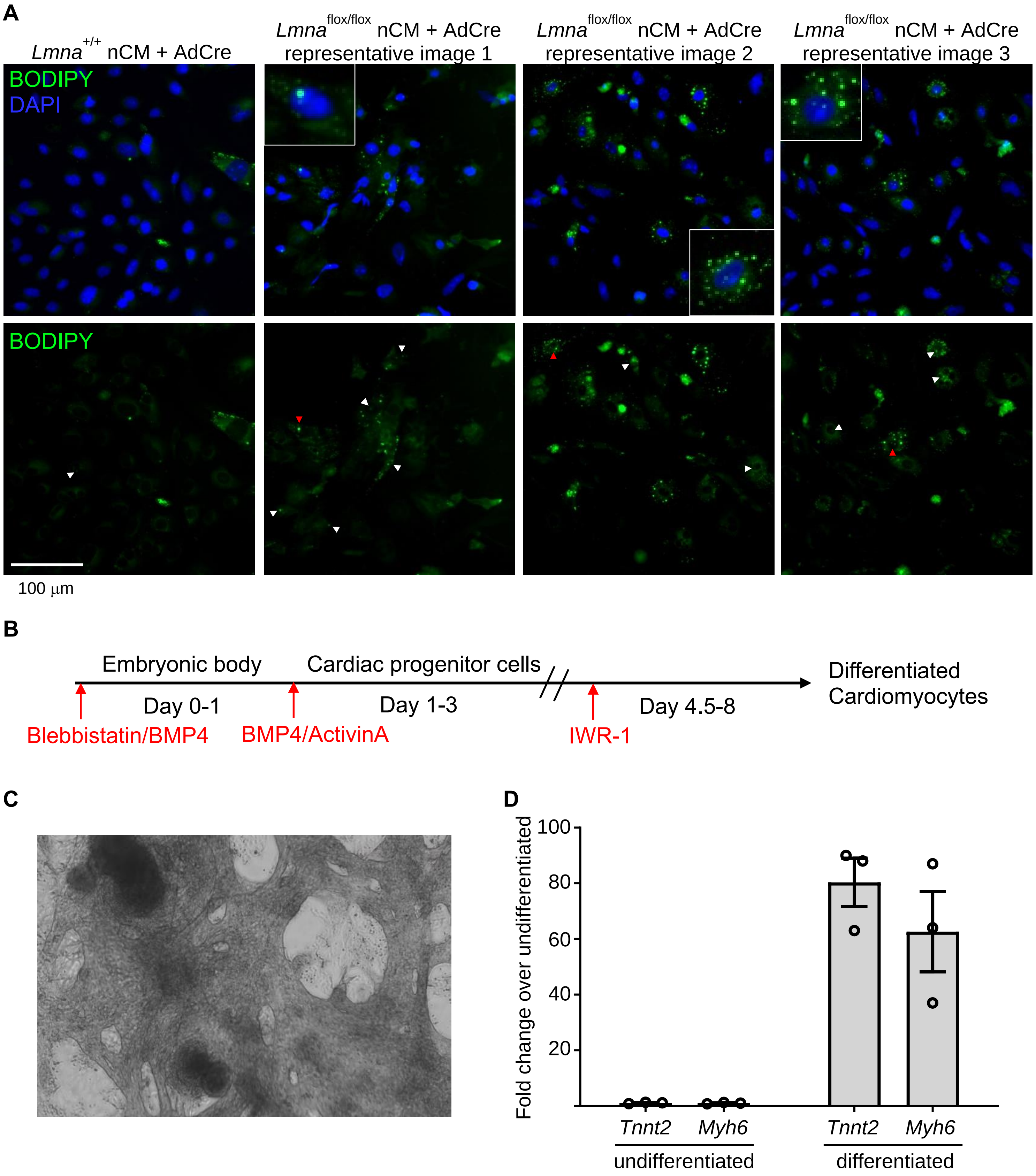

Figure S2. Neonatal CM nuclear lipid droplets and human induced-CM (hiCM) differentiation. A) nCMs isolated from *Lmna*$^{+/+}$ and *Lmna*$^{flox/flox}$ mice infected with AdCre and cultured on 50 kPa matrix for 48 hr after which they were stained with BODIPY 495/503 and DAPI. One representative *Lmna*$^{+/+}$ and three representative *Lmna*$^{flox/flox}$ nCMs images from N = 3 experiments are shown. Scale bar = 100 μm. White arrowheads denote nuclei containing lipid droplets and the red arrowhead denotes magnified nucleus in the inset. **B**) Schematic of the differentiation protocol used to generate hiCMs. **C**) A still image of a video file showing spontaneously contracting control hiCMs. **D**) qPCR on undifferentiated and differentiated iPSC line SCVI114 probed for *Tnnt2* and *Myh6* mRNA. N = 3. Error bars = SEM.

Supplementary Figure S3

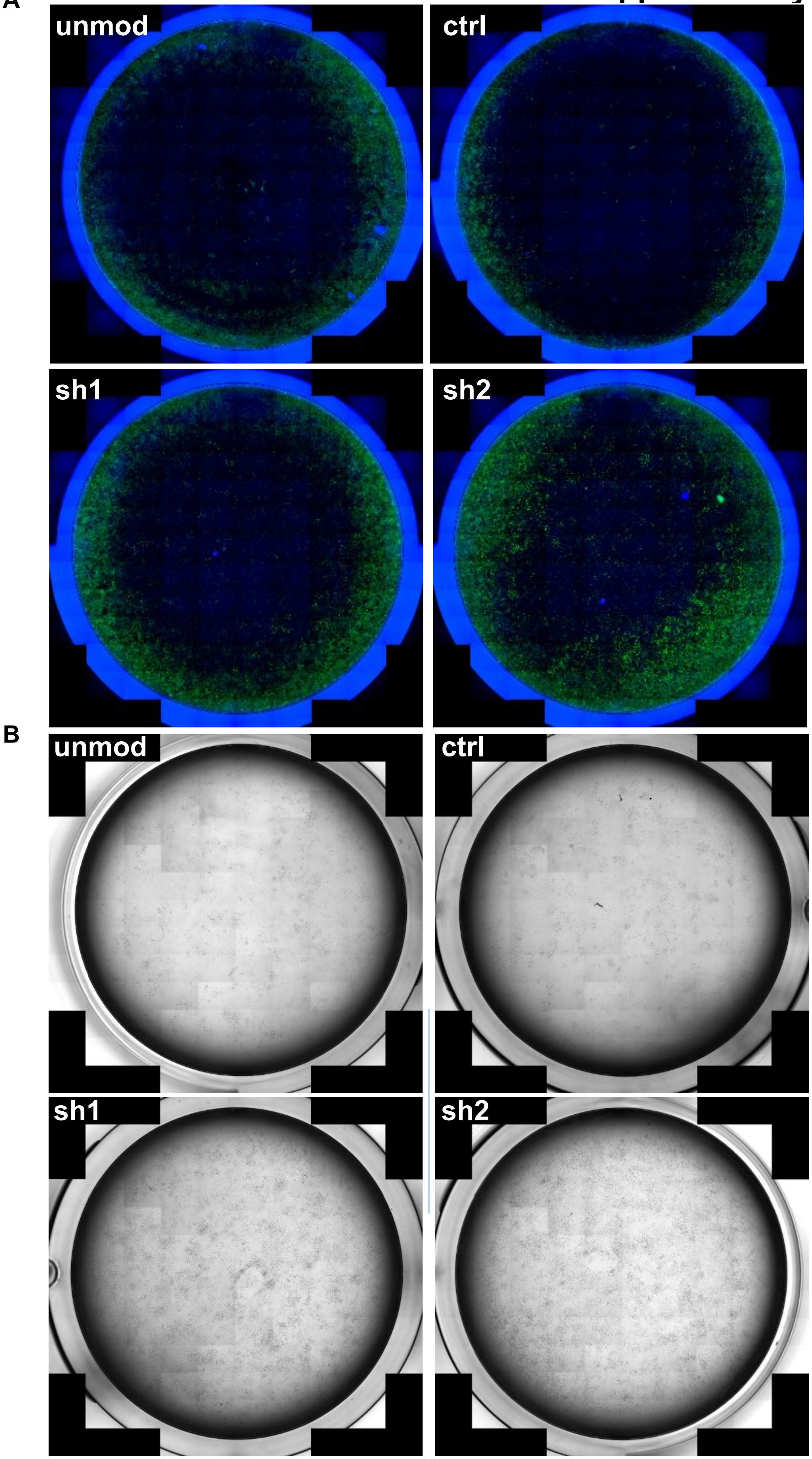

Figure S3. Complete well images of 3T3-L1 differentiated into adipocytes. A) BODIPY and B) brightfield images of the whole well in which *Med25*-silenced 3T3-L1 cells were differentiated in. “unmod” denotes unmodified. A representative images from N = 3 experiments are shown.

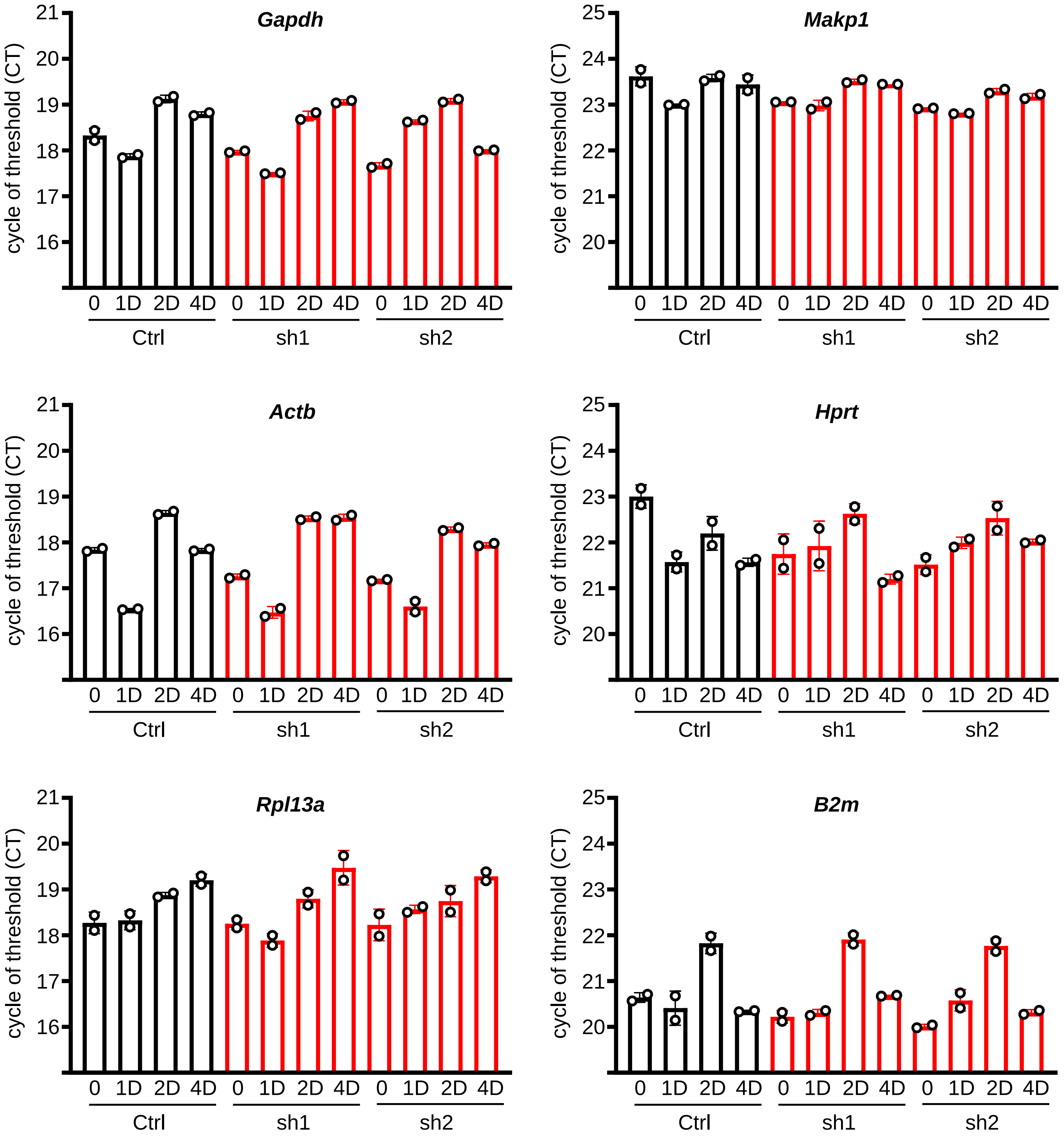

Figure S4. Assessment of internal control gene candidates during 3T3-L1 differentiation. qPCR analyses on mRNA isolated from undifferentiated (0), 1 day (1D), 2 days (2D), and 4 days (4D) post initiation of adipo-genic differentiation in Ctrl, sh1, and sh2 modified 3T3-L1 cells probed for the genes indicated above. Error bars = SEM. N = 2 experiments.

Supplementary Figure S5

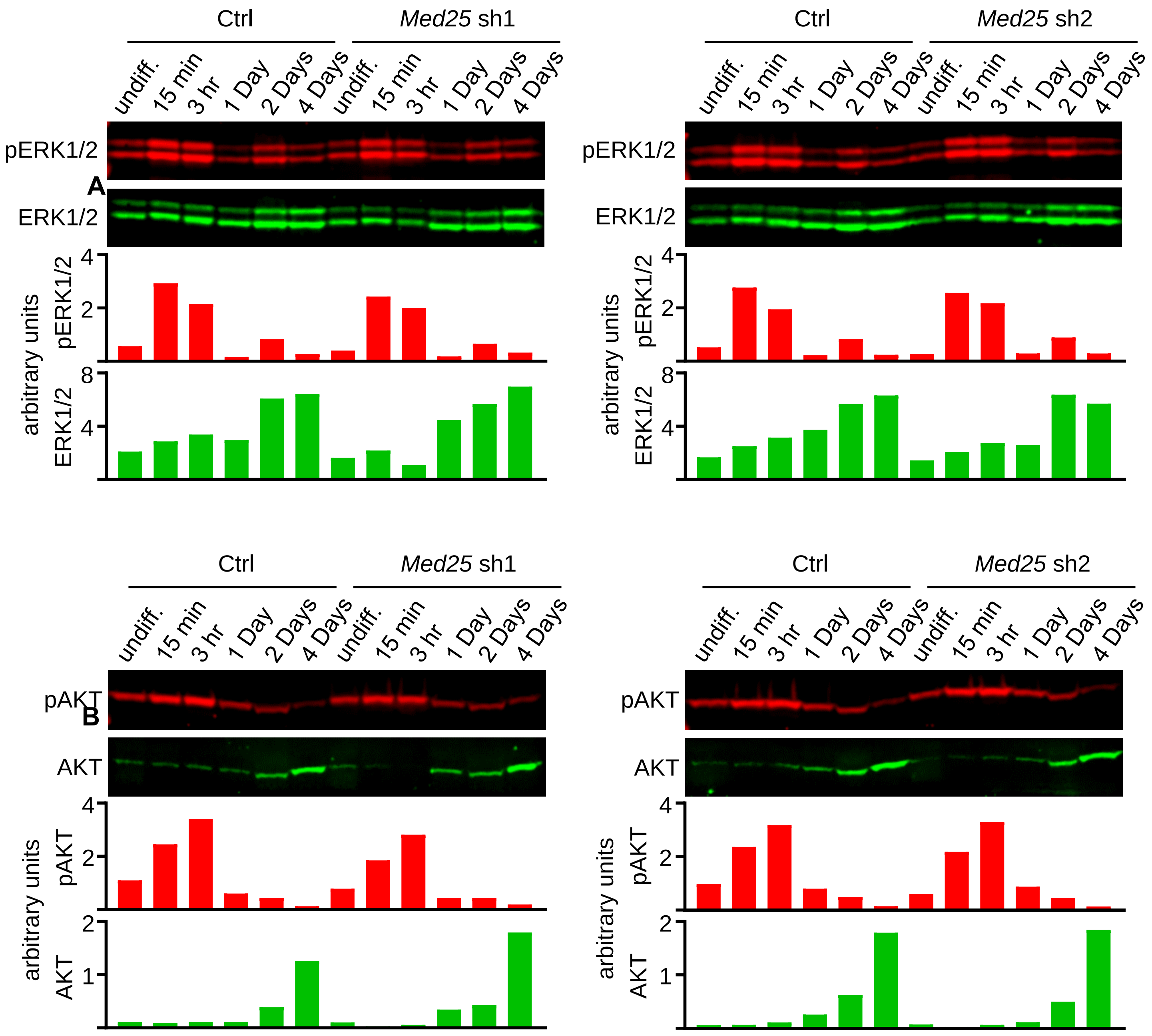

Figure S5. ERK1/2 and AKT activation during adipocyte differentiation in Med25-silenced 3T3-L1 cells. Immunoblot kinetic analyses of total and phosphorylated forms of **A**) ERK1/2 and **B**) AKT with sh1-(left) and sh2-(right) mediated knockdown of Med25. Densitometry measurements are shown below the blots in arbitrary units. Representative blots from N = 3 experiments are shown.

Supplementary Figure S6

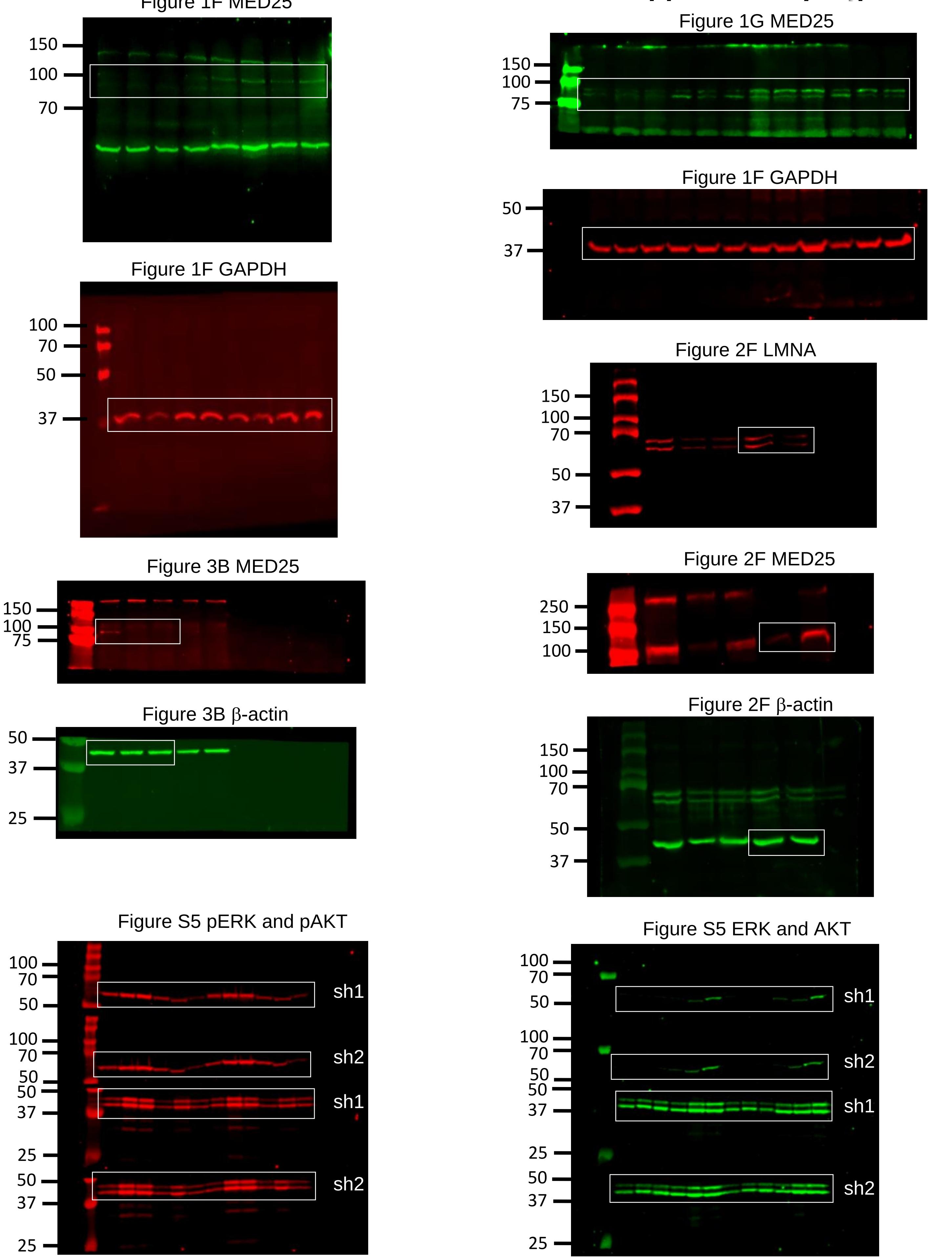

Figure S6. Uncropped immunoblot images.

Supplementary Figure S6

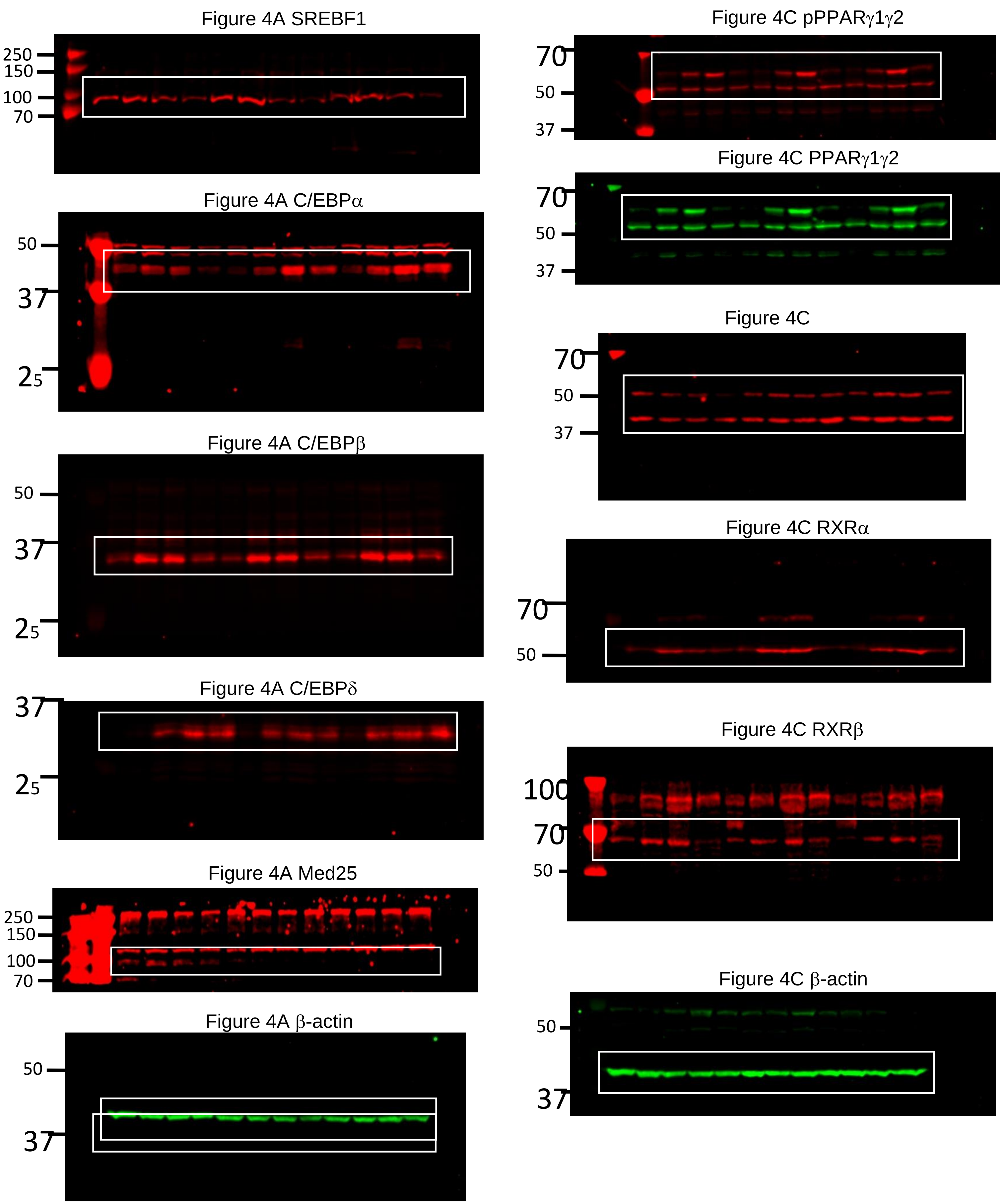

Figure S6. Uncropped immunoblot images continued.

**Primers**	**Forward (5' - 3')**	**Reverse (5' - 3')**
**Mouse**		
Dgat1	GGC TAC TGG GAT CTG AGG TG	CAG GAT CAG CAT CAC CAC AC
Dgat2	CTC TCA GCC CTC CAA GAC AT	CCA CTC CTA GCA CCA GGA AG
Pnpla2 (ATGL)	TCC TCG GGG TCT ACC ACA TT	ATG CAG AGG ACC CAG CAA C
Cd36	TGC TGG AGC TGT TAT TGG TG	TGG GTT TTG CAC ATC AAA GA
Lipe (HSL)	TGG TGA CAC TCG CAG AAG AC	AGT TGG CCT AGG GTT GGT TC
Cpt1b	GTC GCT TCT TCA AGG TCT GG	AAG AAA GCA GCA CGT TCG AT
Mapk1	GGT TGT TCC CAA ATG CTG ACT	CAA CTT CAA TCC TCT TGT GAG GG
Actb	GCT GTA TTC CCC TCC ATC GT	TTC AAT GGG GTA CTT CAG GG
Hprt	GTT GGA TAC AGG CCA GAC TTT GTT G	GAG GGT AGG CTG GCC TAT AGG CT
Rpl13a	ATG ACA AGA AAA AGC GGA TG	CTT TTC TGC CTG TTT CCG TA
B2m	TTC TGG TGC TTG TCT CAC TGA	CAG TAT GTT CGG CTT CCC ATT C
Gapdh	TGC ACC ACC AAC TGC TTA G	GGA TGC AGG GAT GAT GTT C
Pparg1	GAT TTG AAA GAA GCG GTG AAC C	GCA TCT CTG TGT CAA CCA TGG T
Pparg2	TGC CTA TGA GCA CTT CAC AAG AAA T	CGA AGT TGG TGG GCC AGA A
**Genotyping primers**	CTA CGG TGT AAA AGA GGC AGG	CTT GCG AAC CTC ATC ACT CGT
**Human**		
DGAT1	ATC GCC TGC AGG ATT CTT TA	GTC CAC CAG GAT GCC ATA CT
DGAT2	AGT GGG TCC TGT CCT TCC TT	TCT TGG GTG TGT TCC AGT CA
GAPDH	AGG TGG TCT CCT CTG ACT TCA ACA	GAC AAA GTG GTC GTT GAG GGC AAT

Table S1. DNA primers used in the study.

Antibodies	Company	Catalogue #	Concentration<br>IB = immunoblot<br>IF = immunofluorescence
MED25	Santa Cruz Biotechnology	sc-393759	IB(1:500)
Lamin A/C	Santa Cruz Biotechnology	sc-376248	IB(1:2000)
phospho-ERK1/2	Cell Signaling Technology	9102	IB(1:1000)
ERK1/2	Santa Cruz Biotechnology	sc-514302	IB(1:2000)
phospho-AKT	Cell Signaling Technology	4060	IB(1:1000)
AKT	Santa Cruz Biotechnology	sc-81434	IB(1:1000)
phospho-PPARγ (S273)	Bioss	bs-4888R	IB(1:1000)
PPARγ	Perseus Protein Lab	PP-A3409A-00	IB(1:1000)
PPARα	DSHB	PCRP-PPARA-2B9-s	IB(1:50)
C/EBPα	Cell Signaling Technology	2295	IF(1:1000)
C/EBPβ	Cell Signaling Technology	3087	IB(1:1000)
Troponin T	Invitrogen	MA5-12960	IF(1:200)
β-actin	Cell Signaling Technology	3700	IB(1:4000)
GAPDH	Millipore Sigma	MAB374	IB(1:5000)
GFP (TRAP)	Bi-Institutional Antibody and Bioresource Core Facility	HtzGFP_02 (clone 19C8)<br>HtzGFP_04 (clone 19F7)	50ug each per sample
Donkey anti-goat 594	Invitrogen	A-11058	IF(1:400)
Goat anti-mouse 594	Invitrogen	A-21044	IF(1:400)
Goat anti-rabbit 488	Invitrogen	A-11034	IF(1:400)
Goat anti-rabbit 594	Invitrogen	R-37117	IF(1:400)
Licor green mouse	LI-COR Biosciences	926-32210	IB(1:5000)
Licor green rabbit	LI-COR Biosciences	926-32211	IB(1:5000)
Licor red mouse	LI-COR Biosciences	926-68070	IB(1:5000)
Licor red rabbit	LI-COR Biosciences	926-68071	IB(1:5000)

Table S2. Antibodies and their dilutions used in the study.
